# Prescription of selective serotonin reuptake inhibitors in COVID-19 infection needs caution

**DOI:** 10.3389/fpsyt.2022.1052710

**Published:** 2022-10-19

**Authors:** Milica M. Borovcanin, Katarina Vesic, Y. Hasan Balcioglu, Nataša R. Mijailović

**Affiliations:** ^1^Department of Psychiatry, Faculty of Medical Sciences, University of Kragujevac, Kragujevac, Serbia; ^2^Department of Neurology, Faculty of Medical Sciences, University of Kragujevac, Kragujevac, Serbia; ^3^Department of Psychiatry, Bakirkoy Prof Mazhar Osman Training and Research Hospital for Psychiatry, Neurology, and Neurosurgery, Istanbul, Turkey; ^4^Department of Pharmacy, Faculty of Medical Sciences, University of Kragujevac, Kragujevac, Serbia

**Keywords:** SSRIs, COVID-19, antidepressants, inflammation, adverse effect

## Introduction

Although Coronavirus disease (COVID-19) infection is primarily associated with fever, respiratory symptoms, pneumonia, and acute respiratory distress syndrome, the virus causing this infection has shown signs of tropism in other tissues (1). Direct and indirect disturbances affecting the nervous system are well documented, but the exact mechanisms of brain pathology in COVID-19 infection are not fully elucidated (2, 3). Mounting evidence implicates that the novel coronavirus is both neurotropic and vasculotropic (4, 5). Severe acute respiratory syndrome coronavirus-2 (SARS-CoV-2) has the potential to spread directly from the respiratory tract to the central nervous system (CNS) *via* retrograde axonal transport from peripheral nerves (6). In this pathway, nasal inoculation of SARS-CoV-2 led to direct infection of the olfactory epithelium, sensory and olfactory nerves, olfactory bulb and brain (7). An alternative pathway to the CNS is the hematogenous route (8) through which SARS-CoV-2-infected peripheral myeloid cells are transmigrated to organ systems (9). Multi-organ failure is a consequence of the spread of the virus to various organs *via* systemic circulation. Direct endothelial infection leading to disruption of the blood-brain barrier (BBB) or infiltration of immune cells carrying SARS-CoV-2 are presented as possible mechanisms of hematogenous dissemination of the virus (10–12). Angiotensin-Converting Enzyme-2 (ACE-2), a functional receptor of SARS-CoV-2, may facilitate direct invasion of cerebrovascular endothelial cells and neurons, leading to a pro-thrombotic state with occlusion of cerebral vessels, apoptosis, and neuronal cell death (13, 14).

At the onset of the pandemic COVID-19, the medical community made great efforts to establish appropriate treatment protocols and is still advancing in this area. There are a number of newly developed virostatic drugs (15), but another direction has been to use already known agents in these new circumstances. When the use of chloroquine was under discussion, we also discussed the possibility of using chlorpromazine in our previous paper (16). Until the beginning of the pandemic and even today, all psychotropic drugs have been cautiously used in individuals with different types of pulmonary obstruction (17). In light of the emerging fact that infection with COVID-19 is a multisystemic illness associated with various mental disorders, the appropriateness and justifiability of prescribing psychotropic drugs, particularly antidepressants, has been reconsidered. According to the current protocol, there are no precise recommendations for the use of antidepressants in the treatment of COVID-19 infections, especially considering their potential for causal use. In Serbia and Turkiye, only psychiatrists are licensed to prescribe antidepressants, and many consultations have been conducted in intensive care units, with unspoken pressure from colleagues who specialize in somatic treatment that antidepressants should be prescribed urgently to immediately resolve ongoing mental health problems.

We attempt to form our own opinion with clear boundaries, outlining why and when selective serotonin reuptake inhibitors (SSRI) such as fluoxetine, sertraline, fluvoxamine, paroxetine, citalopram, escitalopram, etc. can and should be prescribed in the treatment of COVID-19 infections. Our conclusions and recommendations are based on the recent extensive studies on the use of SSRIs in the treatment of COVID-19 and the known previous data on their mechanism of action, interactions, and prescription in an appropriate time and indication range. As their name implies, the predominant mechanism of action is selective serotonin reuptake inhibition, taking into account the monoaminergic hypothesis, while also expressing their effects on noradrenaline, dopamine, and other neurotransmitter systems. They are indicated and widely used in clinical practice for the treatment of depression (unipolar and bipolar), anxiety disorders, personality disorders, dementia, insomnia, addiction, neuropathic pain, cancer, and even psychosis, etc., and currently SSRI repurposing is very attractive, focusing on immunomodulatory, antiproliferative, and neuroprotective activity (18). Particular attention has been paid to the possible anti-inflammatory properties of SSRIs, which may be useful, although adverse effects have been warned for initial prescription and long-term use.

## Anti-inflammatory properties of selective serotonin reuptake inhibitors in COVID-19 treatment

Most evidence suggests that aberrations in immune-inflammatory pathways contribute to the pathophysiology of depression (19). *Leonard*, in his review article (20), discusses whether sickness behavior is part of a continuum that develops into major depression or whether it is a separate process. Sickness behavior tends to be a short-term response to an acute inflammatory challenge, and when inflammation becomes chronic, mood symptoms predominate and may even worsen the outcome. Depression in COVID-19 infection could be due to viral infection or host immune response (21). Common underlying pathophysiological mechanisms of COVID-19 infection and depression could be the presence of ACE-2 receptors on the cell surface and cytokine secretion (22). More precisely, decreased ACE-2 action and increased production of mediators such as IL-6, TNF-α, and IFN-γ could contribute to depression.

Immune cells seem to have the potential for synthesis, transport, and storage of serotonin but are also responsive to serotonin impact (23). The results of meta-analysis of major depressive disorder studies showed that antidepressants in general and SSRIs and serotonin-norepinephrine reuptake inhibitors (SNRIs) in particular decreased plasma levels of several proinflammatory cytokines such as IL-6, IL-10, TNF-α, C-reactive protein, and C–C Motif Chemokine Ligand (24, 25). A recent meta-analysis revealed that respondents to antidepressants have lower IL-8 than non-responders (26). In analyzing the anti-inflammatory properties of antidepressants, SSRIs have been shown to more potently inhibit microglial production of TNF-α and nitric oxide (NO) *via* regulation of cyclic adenosine monophosphate (cAMP) signaling compared with SNRIs (27). Another possible mechanism of antidepressants is modulation of the NLRP3-inflammasome complex which has been demonstrated in THP-1 cells stimulated with ATP *in vitro* as well as in animal models of stress-induced depression or in depressed patients. Nine drugs, including paroxetine, fluoxetine, mirtazapine, mianserin, desvenlafaxine, venlafaxine, imipramine, amitriptyline, and agomelatine, induced a significant reduction in inflammasome activation by inhibiting IL-1β and IL-18 (28). Considering that many antidepressants have the ability to modulate immune reactions (25), it is likely that the beneficial effects of mental health medications, including SSRIs, on COVID-19 are partly based on their anti-inflammatory activity.

Tryptophan metabolism and kynurenines are related to inflammation and immunity (29), which are also simultaneously being explored as a possible pathway for antidepressant action (30). Analysis of the metabolome profile of patients infected with SARS-CoV-2 revealed the influence of tryptophan-nicotinamide pathway and cytosine on inflammatory signals and microbiota (31). Furthermore, serum analysis of COVID-19 patients showed altered tryptophan metabolism in the kynurenine pathway, which correlated with levels of IL-6 (32), thus implying that antidepressants might control destructive immune activity by balancing a disturbed tryptophan metabolism.

There are some recent suggestions for nonconventional mechanisms of action of SSRIs that may be of use in the treatment of COVID-19 infection. In preclinical models of inflammation and sepsis, the sigma-1 receptor (SIR-1) has been identified as an essential inhibitor of cytokine production (33), and it has been postulated that sigma receptors may be involved in the neuronal transmission of SARS-CoV-2 (34). Fluvoxamine, an SSRI antidepressant, has been shown to enhance a key cellular anti-inflammatory system by stimulating SIR-1 (35–37). Inhibition of the acid sphingomyelinase (ASM)/ceramide system plays an important role and may explain both the potential antiviral and anti-inflammatory effects of certain antidepressants in COVID-19 (38). ASM is an enzyme that converts sphingomyelin to phosphorylcholine and ceramide, and high concentrations of ceramide in the cell membrane are thought to disrupt membrane integrity, thereby facilitating viral entry (39, 40). The magnitude of *in vitro* inhibition of ASM by SSRIs varies across molecules (e.g., fluoxetine > paroxetine > fluvoxamine > other SSRIs) (41, 42), and appears to correlate with the magnitude of *in vitro* antiviral effect against SARS-CoV-2 (43, 44). Fluoxetine has been shown to inhibit SARS-CoV-2 entry into epithelial cells as well as SARS-CoV-2 replication (43, 45, 46). Moreover, decreased immunoglobulin E-mediated mast-cell degranulation and impaired endolysosomal viral trafficking are considered mechanisms limiting hyperinflammatory immune responses (47–49). The antiviral activity of SSRIs could also be reflected in targeting phospholipid production and melatonin levels (50).

## Somatic benefits of selective serotonin reuptake inhibitors prescription for COVID-19 treatment

The newest clinical experience in the treatment of COVID-19 has demonstrated the beneficial role of antidepressant use, particularly SSRIs, in somatic status and potential outcomes. More importantly, however, this benefit has been observed not only for mental functioning but also for the somatic state of these patients. In exploring the “anti-COVID-19” potential of antidepressants, SSRIs such as fluvoxamine and, to a lesser extent, fluoxetine have been shown to be the most important drugs with positive effects on overall disease outcome.

Application of fluvoxamine as an additional treatment for COVID-19 significantly reduced severe COVID-19 outcomes and effectively prevented clinical worsening and hospitalization (51, 52), and also reduced mortality in COVID-19-patients hospitalized in the intensive care unit (53). Furthermore, it predicted fewer hospitalizations and residual symptoms in SARS-CoV-2-positive adult home-isolated patients (54). In a recent study, fluvoxamine was examined at a low dose of 50 mg twice daily but did not prevent hypoxia, emergency department visits, hospitalization, or death (55).

The use of SSRIs, particularly fluoxetine, was associated with a lower relative risk of death compared with patients not on SSRIs (56). The protective effect of antidepressants in COVID-19 was mainly governed by SSRI, SNRI, and serotonin-2 antagonist reuptake inhibitors (57). Adult patients hospitalized with moderate or severe COVID-19 pneumonia that received fluoxetine along with anti-COVID-19 therapies had significantly decreased mortality (70%) compared to the non-fluoxetine group (58). Exposure to fluoxetine, venlafaxine, mirtazapine, and escitalopram was found to be significantly associated with a lower risk of intubation or death in COVID-19 patients (59).

On the other hand, no relevant influence of antidepressants on COVID-19 duration and severity was observed (60). The meta-analyses by Vai et al. (61) showed an increased risk of COVID-19 mortality in patients with psychotic and mood disorders, and those taking antipsychotics or anxiolytics represent a susceptible subgroup, whereas antidepressant use had no effect on mortality risk. No significant difference in mortality risk was observed between patients taking/non-taking SSRIs (62). The use of tricyclic and related antidepressants prior to COVID-19 diagnosis was not associated with the occurrence of severe COVID-19 clinical symptoms (63).

Patients with severe mental illness (e.g., schizophrenia spectrum disorder, bipolar disorder, unipolar depression) have been shown to be at increased risk for a more drastic COVID-19 course and resulting lethality (64–66). According to recent evidence, this risk may not be due to the psychiatric illness alone, but rather to the presence of somatic comorbidity, which significantly influences the development of COVID-19 morbidity and mortality, especially in the specific population of psychiatric patients (67, 68). On the basis of these data, we strongly recommend psychiatric examination and safety assessment before the application of SSRIs in COVID-19 patients, regardless of their mental illness history.

## Obstacles to selective serotonin reuptake inhibitors prescription in COVID-19 treatment

We must point out that it is necessary to continue treatment with antidepressants in patients with mental disorders, even if they have contracted SARS-CoV-2. There are no data indicating absolute contraindications in these cases. The inclusion of known SSRI antidepressants in the treatment protocols of COVID-19 requires more thorough consideration and precautions, as potential risks may arise. It is well known that these drugs can cause adverse side effects despite their good safety profile, even when used for the right indications. Expected somatic side effects such as nausea and abdominal discomfort, diarrhea and vomiting, headache, insomnia, drowsiness, and dry mouth may also occur (69).

Due to their potent anti-depressive effects, SSRIs can induce a manic switch or mixed episode or a long-term condition called rapid cycling in patients with undiagnosed bipolar affective disorder (70). On the other hand, ample evidence suggests that SARS-CoV-2, by its very nature, can also trigger the acute onset of mood disorders or psychotic symptoms (71), further complicating the prescription of SSRIs. After the first week of treatment with SSRI, clinical improvement was observed with an increased chance of a 50% reduction in Hamilton Depression Rating Scale scores at 1 week compared to placebo (72). Dionisie et al. (73) discuss that the clinical effect of SSRIs occurs after 2–4 weeks of treatment, suggesting that it is not only the increase in monoamine transmitters that is responsible for the improvement in depressive symptoms. All this points to the previous properties of SSRIs in inflammation, infection, and neuroprotection, and subsequently we need to be concerned about their effects on serotonergic transmission, especially in individuals without diagnosed depression.

In patients with COVID-19, the use of antidepressants may be particularly challenging since these medications may interact with medical treatments for COVID-19 and some of their adverse effects may worsen the course and outcome of the underlying medical condition. It is extremely important to consider the interactions of SSRIs with antiviral and other COVID-19 medications (74, 75) to determine how meaningful their repurposing is in fighting this infection. Because most COVID-19 patients are treated with low-molecular-weight heparin or novel peroral anticoagulants, it is very important to monitor hemorrhagic side effects. This is especially important when SSRIs are administered concomitantly, as SSRIs have been shown to affect platelet function (76).

## Discussion

**Figure 1:**
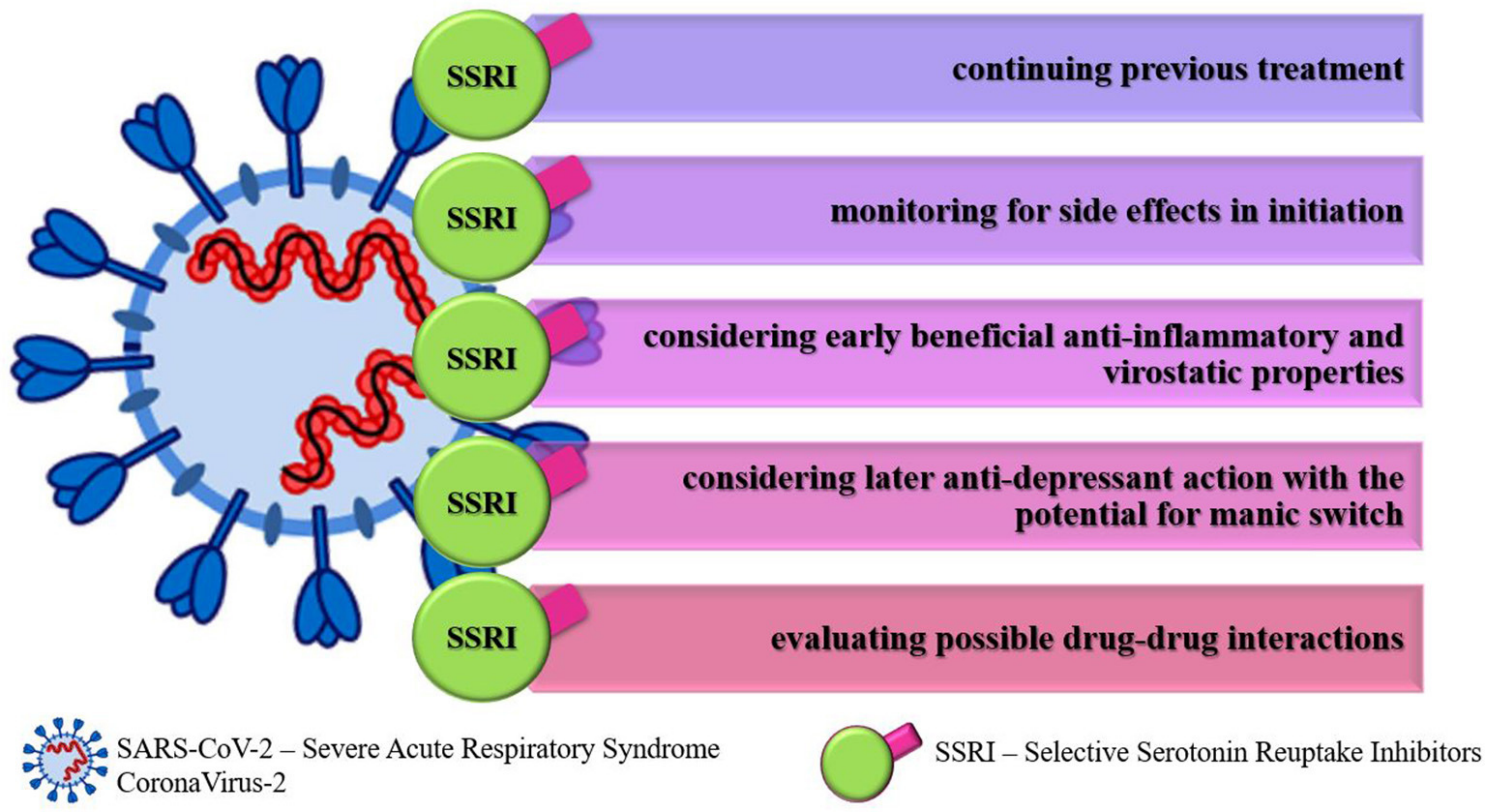
Recommendations for selective serotonin reuptake inhibitors usage in COVID-19 infection.

Although the benefits of mental health medications, such as SSRIs in coronavirus infection have been documented to date, we have attempted to provide a balanced review of current knowledge and the risks associated with the unwarranted and imprudent clinical use of antidepressants in patients with COVID-19 infection. These drugs should certainly be applied with more caution and consideration. In addition, COVID-19 patients undergoing SSRI treatment should be closely monitored for possible adverse effects (all summarized in Figure 1).

As the SARS-CoV-2 virus becomes more controllable, its pandemic importance is diminishing, necessitating a revision of therapeutic protocols. Currently, ongoing clinical trials are expected to exclude some drugs, while new drugs will improve the drug repository. We believe that further clinical studies will demonstrate determinately whether the use of these antidepressants in COVID-19 is efficient and clinically justified.

## Author contributions

All authors listed have made a substantial, direct, and intellectual contribution to the work and approved it for publication.

## Conflict of interest

The authors declare that the research was conducted in the absence of any commercial or financial relationships that could be construed as a potential conflict of interest.

## Publisher's note

All claims expressed in this article are solely those of the authors and do not necessarily represent those of their affiliated organizations, or those of the publisher, the editors and the reviewers. Any product that may be evaluated in this article, or claim that may be made by its manufacturer, is not guaranteed or endorsed by the publisher.
